# Effect of ageing on CMV-specific CD8 T cells from CMV seropositive healthy donors

**DOI:** 10.1186/1742-4933-6-11

**Published:** 2009-08-28

**Authors:** María Luisa Pita-Lopez, Inmaculada Gayoso, Olga DelaRosa, Javier G Casado, Corona Alonso, Elisa Muñoz-Gomariz, Raquel Tarazona, Rafael Solana

**Affiliations:** 1 University of Cordoba, Department of Cellular Biology, Physiology and Immunology, Faculty of Medicine, Cordoba, Spain; 2 University of Extremadura, Department of Physiology, Immunology Unit, Caceres, Spain; 3 Research Methodology Unit, H. U. Reina Sofia, Cordoba, Spain; 4 Molecular Biology and Immunology Unit, Department of Health and Well-being, South University Center, University of Guadalajara, Guzman City, Jalisco, Mexico

## Abstract

**Background:**

Ageing is associated with changes in the immune system with substantial alterations in T-lymphocyte subsets. Cytomegalovirus (CMV) is one of the factors that affect functionality of T cells and the differentiation and large expansions of CMV pp65-specific T cells have been associated with impaired responses to other immune challenges. Moreover, the presence of clonal expansions of CMV-specific T cells may shrink the available repertoire for other antigens and contribute to the increased incidence of infectious diseases in the elderly. In this study, we analyse the effect of ageing on the phenotype and frequency of CMV pp65-specific CD8 T cell subsets according to the expression of CCR7, CD45RA, CD27, CD28, CD244 and CD85j.

**Results:**

Peripheral blood from HLA-A2 healthy young, middle-aged and elderly donors was analysed by multiparametric flow cytometry using the HLA-A*0201/CMV pp65_495–504 _(NLVPMVATV) pentamer and mAbs specific for the molecules analysed. The frequency of CMV pp65-specific CD8 T cells was increased in the elderly compared with young and middle-aged donors. The proportion of naïve cells was reduced in the elderly, whereas an age-associated increase of the CCR7^null ^effector-memory subset, in particular those with a CD45RA^dim ^phenotype, was observed, both in the pentamer-positive and pentamer-negative CD8 T cells. The results also showed that most CMV pp65-specific CD8 T cells in elderly individuals were CD27/CD28 negative and expressed CD85j and CD244.

**Conclusion:**

The finding that the phenotype of CMV pp65-specific CD8 T cells in elderly individuals is similar to the predominant phenotype of CD8 T cells as a whole, suggests that CMV persistent infections contributes to the age-related changes observed in the CD8 T cell compartment, and that chronic stimulation by other persistent antigens also play a role in T cell immunosenescence. Differences in subset distribution in elderly individuals showing a decrease in naive and an increase in effector-memory CD8 T cells may be relevant in the age-associated defective immune response.

## Background

Human cytomegalovirus (CMV) infection in immunocompetent individuals is normally asymptomatic, but can be a major cause of morbility in immunosuppressed individuals. After primary infection the virus persists throughout life in a latent form in a variety of tissues, particularly in precursor cells of the monocytic lineage [1]. Host defence against infection by CMV is ensured in great part by cytotoxic CD8 T lymphocytes directed against the tegument protein pp65 [2]. In immunosuppressed, and occasionally immunocompetent persons, CMV can be reactivated and, in these situations, the presence of CMV-specific CD8 T cells which are not producing IFNγ, and therefore potentially anergic or in vivo exhausted is frequent [3].

Ageing is associated with changes in the immune system with substantial alterations in T-lymphocyte subsets. CMV infection substantially modulates the peripheral lymphoid pool in healthy donors [4,5]. CMV also affects functionality of T cells and the differentiation and large expansion of CMV-specific T cells have been associated with impaired responses to other immune challenges [6]. Moreover, clonal expansions of CMV-specific T cells may shrink the available repertoire for other antigens and contribute to the increased incidence of infectious disease in the elderly [7-10]. In aged people, the CMV phosphoprotein pp65 (UL83) is the major antigen recognised by T lymphocytes targeting functionally efficient T cell effector responses with massive production of Th1 cytokines and exhibition of CD107a degranulation marker [11]. The percentage of these cells are strikingly expanded and the great majority are CD28^-^[12,13]. In addition the CD8 T cell subset shows a significant increase of the CD45RA^+ ^CD27^- ^subset, likely as a consequence of acute CMV infection [14].

The pool of memory T cells functions as a dynamic repository of antigen-experienced T lymphocytes that accumulate over the individual's lifetime. Several subpopulations of human CD8 T cells are defined according to the expression of CCR7, CD45RA and cytolytic effector molecules [15-17]. The expression of CCR7 divides human memory T cells into two functionally distinct subsets with distinct homing capacity and effector function [15,16]: those expressing CCR7 are termed central memory (CM), whereas effector memory (EM) cells are characterised by the lack of CCR7. Whereas CM CD8 T cells are phenotypically homogeneous, different EM subpopulations have been defined within the CCR7^null ^subset according to the expression of different markers including the level of expression of CD45RA or the expression of the co-stimulatory molecules CD27 and CD28 [18-20].

In vitro senescence models and cross-sectional *ex vivo *studies have consistently demonstrated that senescent T cells and T cells from aged individuals express unusually high densities of receptors that are normally found on natural killer (NK) cells [21,22]. On CD8 T cells, the expression of NK associated receptors increases with age whereas expression of CCR7 decreases [23]. CD85j (also called LIR-1/ILT2/LILRB1), is an inhibitory cell surface receptor expressed on NK cells, B lymphocytes, monocytes, dendritic cells, and T cell subsets. CD85j recognises a broad range of classical and non-classical MHC class I molecules and CD85j triggering inhibits T cell function [24,25]. In contrast, CD85j interaction with UL18, an MHC class I homologue encoded by human cytomegalovirus, leads to activation of T cells, resulting in lysis of CMV-infected cells [26,27]. The expression of CD85j has been correlated with CD8 T-cell differentiation into effector cells and it is increased on virus-specific CD8 T cells in chronic infection [25]. CD244 (2B4) is a transmembrane receptor of the Ig superfamily primarily expressed by NK cells and antigen-experienced CD8 T cell subsets [28,29]. It is required for optimal activation of CD8 T cells and NK cells [30] playing an important role in activating cytotoxicity through its interaction with CD48 on some target cells [28,29,31].

**Figure 1:**
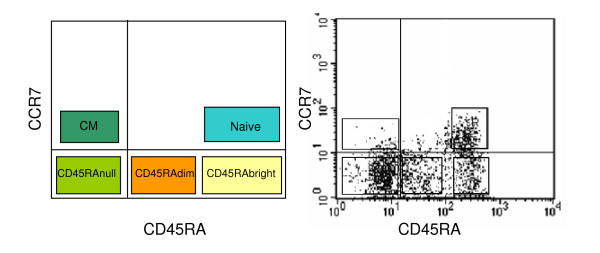
**CD8 T cell-subset distribution according to the expression of CCR7 and CD45RA**. Schematic representation (left) and a representative experiment (right) of the five CD8 T cell subsets that can be defined by the analysis of CCR7 and CD45RA markers.

In this study, we analyse the effect of ageing on the frequency of total and CMV-specific CD8 T cell subsets defined by the expression of CCR7 and CD45RA. Using these markers we have defined naïve (CCR7^+ ^CD45RA^bright^), CM (CCR7^+ ^CD45RA^nulls^) and three subpopulations of EM CD8 T cells, characterised by the absence of CCR7, and defined according to the level of expression of CD45RA as EMRA^null^, EMRA^dim ^and EMRA^bright ^(Figure 1). The expression of CD27, CD28 and the NK cell-associated markers CD85j and CD244 on these subsets is also studied.

## Results

### The frequency of CMV pp65-specific CD8 T cells is increased in healthy elderly when compared with young and middle aged individuals

**Figure 2:**
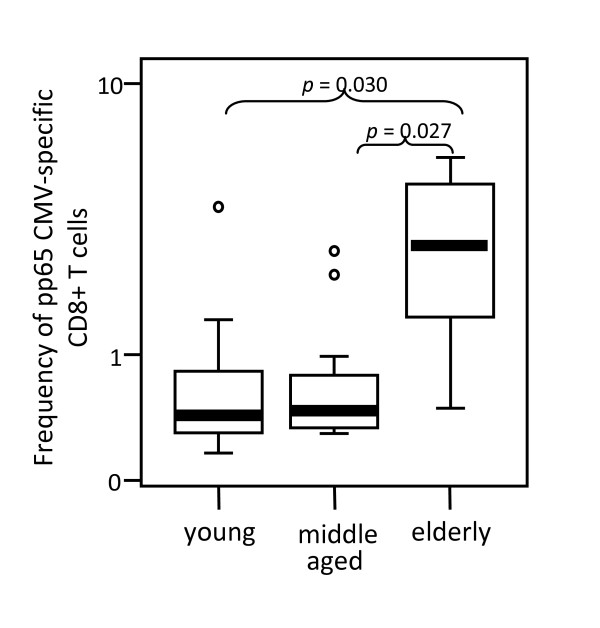
**Elderly individuals show an increased frequency of CMVpp65-specific CD8 T cells**. The frequency of A2/CMVpp65 pentamer-positive CD8 T cells was analysed in young, middle aged and elderly donors. Data are presented as diagrams of boxes; the lower boundary of the box indicates the 25th percentile and the upper boundary the 75th percentile. Error bars above and below the box indicate the 90th and 10th percentiles. A line within the box marks the median. Outliers are represented as individual points. P values lower than 0.05 were considered statistically significant.

The analysis of the frequencies of CMV pp65-specific CD8 T cells in HLA-A2, CMV-seropositive donors using A2/CMV-pp65 pentamers is shown in Figure 2. Pentamer-positive CD8 T cells were increased in the elderly compared with young (*p *= 0.03) and middle aged (*p *= 0.027) donors. No significant differences were observed when values from young and middle aged donors were compared.

### Age-associated expansion of effector-memory CMVpp65-specific CD8 T cells

**Figure 3:**
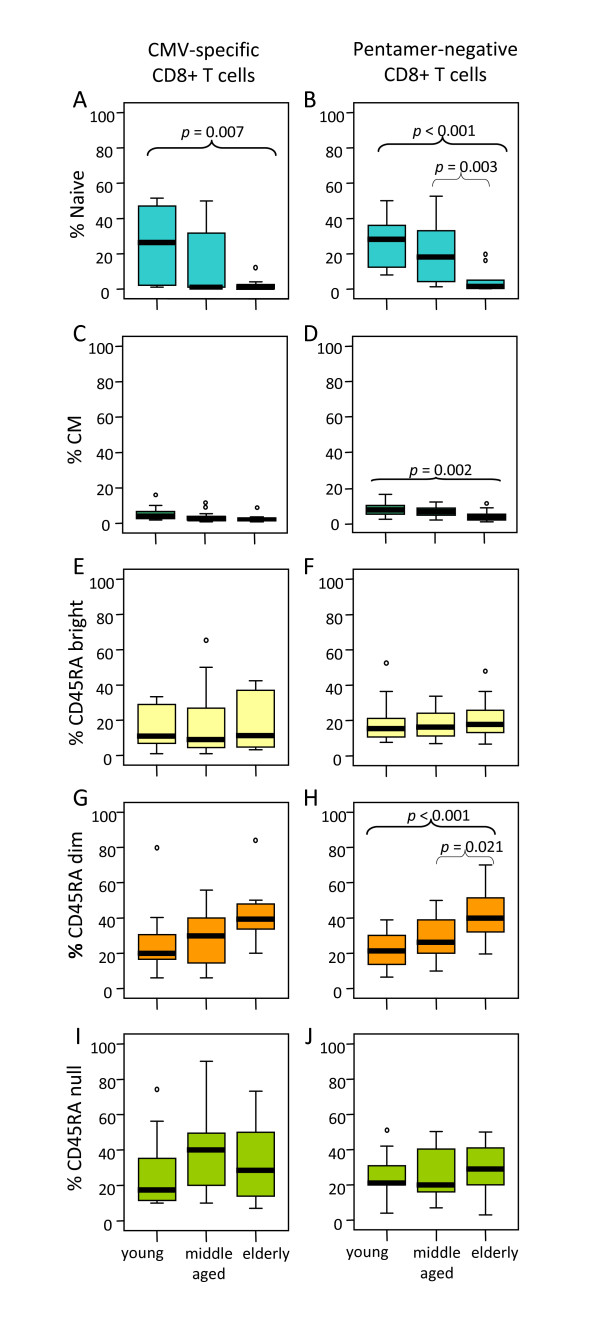
**Influence of age on CD8 T cell subset distribution**. According to the subset model depicted in Figure 1, CD8 T cells were subdivided into naive, CM, EMRA^bright^, EMRA^dim^, EMRA^null ^cells and analysed in young, middle aged and elderly individuals. Results for CMV pentamer-positive (left) and pentamer-negative (right) CD8 T cells are shown. Data are presented as diagrams of boxes as indicated in Figure 2 legend.

Age-associated changes in naive, CM and the three subsets of EM (EMRA^null^, EMRA^dim ^and EMRA^bright^) in pentamer-positive and pentamer-negative CD8 T cells are shown in Figure 3. The results demonstrated that the proportion of naïve A2/CMV-pp65 pentamer-positive CD8 T cells were significantly reduced in elderly individuals compared with young donors (*p *= 0.007) (Figure 3A). No significant changes on CM pentamer-positive CD8 T cells were found (Figure 3C). When pentamer-negative CD8 T cells were analysed an age-associated decrease in the proportion of naïve cells (elderly *vs *young *p *< 0.001 and elderly *vs *middle aged *p *= 0.003) and CM (elderly *vs *young *p *= 0.002), (Figure 3B and 3D) were found, corroborating that the proportions of these CD8 T cell subsets are decreased with age.

In relation with the EM subsets, the results showed, both in the pentamer-positive and pentamer-negative CD8 T cells, an age-associated increase of the percentage of EMRA^dim ^cells (Figure 3G and 3H), whereas no significant differences were found in the EMRA^bright ^(Figure 3E and 3F) or in the EMRA^null ^(Figure 3I and 3J) subsets. No significant differences in the distribution of naïve/memory CD8 T cell subsets have been found when comparing pentamer-positive and pentamer-negative CD8 T cells in each age group.

### Decreased expression of CD27 and CD28 on the EM CD8 T cell subsets in the elderly

**Figure 4:**
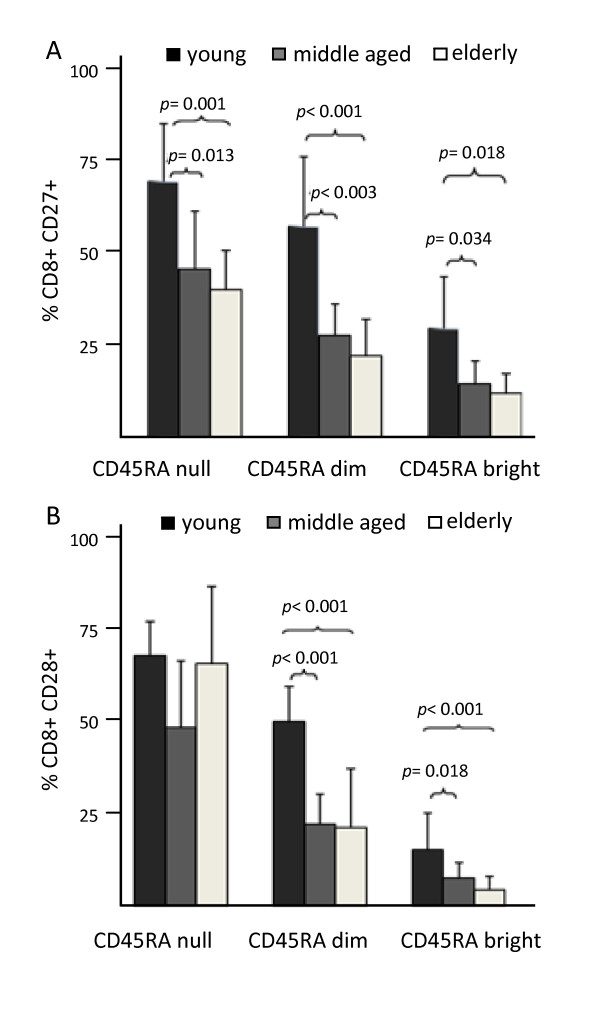
**Expression of CD27 and CD28 on the effector-memory CD8 T cell subsets**. EM CD8 T cells defined as CCR7^null ^were subdivided into further subsets according to the model in Figure 1 and analysed in young, middle aged and elderly individuals. Columns and bars represent the mean and SD.

In order to further characterise age-associated changes in the CCR7^null ^EM T cell subsets, the expression of CD27 and CD28 was analysed. The results showed that the EMRA^null ^subset had a higher expression of both CD27 and CD28 markers than the EMRA^dim ^and EMRA^bright ^subsets in the groups of young, middle age and elderly donors. Furthermore, a decreased expression of CD27 was observed in the middle age and elderly group compared with young individuals in the three EM subsets considered (Figure 4A). A similar age-associated decrease was observed in the expression of CD28 in the EMRA^dim ^and EMRA^bright ^subsets, whereas no significant age-associated differences were found in the expression of CD28 within the EMRA^null ^subset (Figure 4B).

### Most A2/CMV-pp65 pentamer-positive CD8 T cells from elderly individuals are CD27 and CD28 negative cells expressing CD85j and CD244

**Figure 5:**
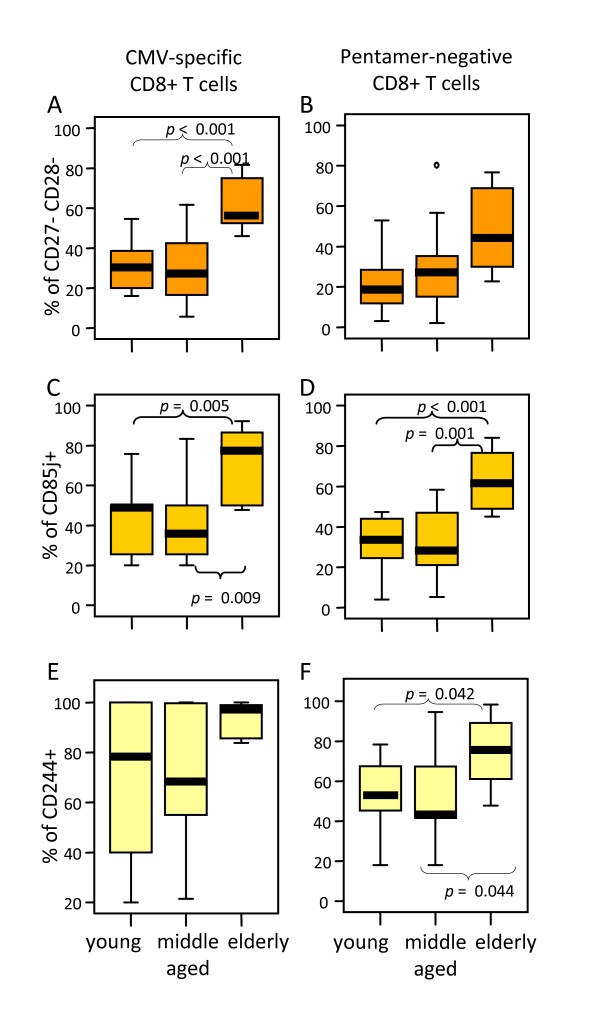
**The majority of A2/CMV-pp65 pentamer-positive CD8 T cells from elderly individuals display a CD27^- ^CD28^- ^phenotype and express CD85j and CD244**. The percentage of CD27^-^CD28^- ^(A), CD85j^+ ^(B) and CD244^+ ^(C) CD8 T cells was analysed on PBMC gated on CMV pentamer-positive (left) and pentamer-negative (right) CD8 T cells in young, middle aged and elderly donors. Data are presented as diagrams of boxes as indicated in Figure 2 legend.

The analysis of CD27 and CD28 expression in CMV pp65-specific CD8 T cells demonstrated that elderly individuals had an increased proportion of CD27^-^CD28^- ^cells compared with young (*p *< 0.001) and middle aged (*p *< 0.001) donors (Figure 5A). Although, an age-associated increase of CD27^-^CD28^- ^cells was also found in A2/CMV-pp65 pentamer-negative CD8 T cells, no significant statistical differences were found (Figure 5B).

**Figure 6:**
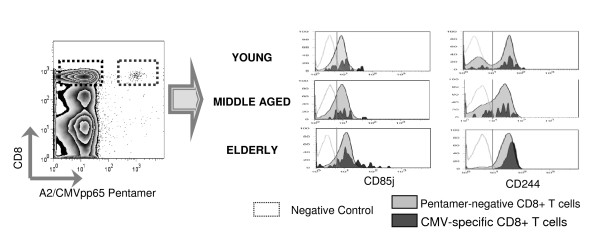
**Analysis of CD85j and CD244 expression on CD8 T cells**. Dot-plot chart illustrates how pentamer-positive and pentamer-negative CD8 T cells were selected. Histograms show the expression of CD85j and CD244 in A2/CMVpp65 pentamer-positive (dark grey) and pentamer-negative (light grey) CD8 T cells from representative young, middle-aged and elderly individuals. The empty histograms represent the negative isotype control for each experiment.

To further characterise the phenotype of CMV pp65-specific CD8 T cells we have analysed the expression of CD85j, a receptor for the human CMV MHC class I homologue UL18. Our results showed an increased expression of CD85j both in A2/CMV-pp65 pentamer-positive and pentamer-negative CD8 T cells from elderly individuals when compared with young and middle aged donors (Figure 5C and 5D). An age-associated increase in the expression of CD244 on CD8 T cells was also found in pentamer-negative CD8 T cells. Statistically significant differences were observed when elderly donors were compared with young (*p *= 0.044) and middle aged (*p *= 0.042) individuals whereas no statistical significant differences were found in the expression of CD244 when CMV pp65-specific T cells were considered (Figure 5E and 5F). Representative flow cytometry analyses of the expression of CD85j and CD244 in pentamer-positive and negative CD8 T cells from young, middle-aged and elderly individuals are shown in Figure 6.

## Discussion

In this work the effect of ageing on the frequency and phenotype of CMV-specific CD8 T cell subsets has been studied. Increasing evidences support that many of the alterations observed in the T cell compartment in the elderly can be the consequence of chronic activation of the immune system by latent virus such as CMV [9,10,13,32-40]. Thus remodelling of the CD8 T cell compartment in the elderly, characterised by a decrease of naive cells and increasing numbers of cells with a memory/activated phenotype [13,41-48], could be a consequence not only of thymic involution (changes related to age) but also due to CMV chronic antigenic stimulation as recently indicated [4,5,10,13,49].

An increased frequency of CMV-specific CD8 T cells was observed in the elderly compared to young and middle aged individuals. Our results on the analysis of the effect of ageing on the phenotype of CMV-specific CD8 T cells show that elderly individuals have a decrease of A2/CMVpp65 pentamer-positive CD8 T cells with a naive phenotype and an increase of those with an EM phenotype, compared with young individuals [10,13]. The increase in the EM subset is mainly due to the expansion of those A2/CMVpp65 pentamer-positive CD8 T cells displaying the EMRA^dim ^phenotype, whereas the percentage values of EMRA^bright ^and EMRA^null ^CD8 T cells were similar in young, middle aged and elderly donors.

The CD8 T cell compartment as a whole showed a decreased frequency of naive and CM cells and an increase of the EMRA^dim ^CD8 T cell subset. These results confirm previous studies showing a dramatic decrease in the percentage of naive and the expansion of EM CD8 T cells with age [10,48,50]. The decreased percentage of naive CD8 T cells (defined as CCR7+ CD45RA+ CD28+ CD27+) and the expansion of EM CD8 T cells, showing a wide range of phenotypes [19], have been used in several studies as biomarkers of immunosenescence [38,40,48]. A recent study that analyses age-associated changes in CD8 cells in CMV seronegative and seropositive individuals strongly support that CMV is the predominant stimulus for the generation of CD45RA+ EM CD8 cells [4]. However, our results showing that there are not remarkable differences in the naïve/and different memory T cells between A2/CMV-pp65 -specific and non specific CD8 T cells, suggest that this is not the unique chronic stimulus as previously demonstrated in the northern European population and in CMV-negative elderly donors [4,5,7,51,52].

The expression of the CD28^- ^phenotype is a characteristic of replicative senescence [53,54]. Chronic antigenic stimulation has been associated with peripheral blood expansions of CD28^- ^CD8 T cells also characterised by loss of CD27 and expression of NK cell-associated markers as CD57 [22,28,55-57]. We observed an increase of CMV-specific CD8 T cells displaying a CD27^-^CD28^- ^phenotype in the elderly suggesting that clonal expansion of T cells in response to chronic antigenic stimulation results in the accumulation of senescent T cells. In the pentamer-negative CD8 T cells a similar tendency was observed in the expression of CD27 and CD28 although no significant differences were observed between young, middle aged and elderly individuals.

CD85j is an inhibitory cell surface receptor that has high affinity for UL18, an MHC class I homologue encoded by human cytomegalovirus. Despite the well defined inhibiting functions of CD85j, its interaction with UL18 leads to activation of T cells, resulting in lysis of CMV-infected cells [26,27]. CD85j expression has been correlated with CD8 T-cell differentiation into effector cells [25]. An age-associated increase of CD85j on CD8 T cells has been reported that correlates with CD28 down-regulation and CD57 up-regulation [23,58,59]. Here we show that elderly individuals have an increased expression of CD85j within both CMV-specific and pentamer-negative CD8 T cells. Thus, its expression could be used as an indicator of the encounter with CMV pp65 epitope presented by the APCs to CD8 T cells. In addition, CD85j expression could be a used as a marker of replicative senescence [23,59], in parallel with the CD27^- ^CD28^- ^phenotype or CD57 expression. CD85j^+ ^CD27^- ^CD28^- ^CMV pp65-specific CD8 T cells were increased in the elderly compared with young and middle aged suggesting that CMV-specific CD8 T cells have undergone extensive rounds of CMV antigen-driven stimulation reaching replicative senescence. The expression of CD244, a marker of T cell differentiation into effector cells [60,61], was found elevated in CMV-specific CD8 T cells compared to whole CD8 T cells.

Similar frequencies of CMV-specific CD8 T cells and T cell subsets defined by CCR7 and CD45RA can be found in young and middle-age donors. The high variability observed in the middle aged population could be related to the time point of CMV infection or the different immune response from each individual to virus. It should be interesting to know the evolution of those individuals in this age-group. It can be speculated that chronic activation of CMV-specific CD8 T cells in young and middle aged donors might have detrimental effects on age-associated diseases in this population.

## Conclusion

It has been demonstrated that naturally occurring CD4 and CD8 T cell responses to pp65 were detectable in many subjects [62]. Phenotypic characterization of CMV-specific CD8 T cells can benefit the development of adoptive therapies and vaccination protocols. The finding that the CMV-specific CD8 T cell phenotype in elderly individuals is similar to the predominant phenotype of CD8 T cells as a whole, suggest that latent infection with CMV can be considered as a major force contributing to the differentiation of CD8 T cells into CD27^- ^CD28^- ^cells. These data confirm that the chronic antigenic stimulation induced by persistent viral life-long infections like CMV may induce important changes in the CD8 T cell compartment.

## Donors, materials and methods

### Subjects

Peripheral blood from healthy donors was obtained after informed consent under the auspices of the appropriate Research and Ethics Committees. Eighteen healthy young individuals (age range, 21 to 40 years; mean ± standard deviation (SD), 29 ± 6 years), seventeen healthy middle aged donors (age range, 41 to 64 years; mean ± SD, 51 ± 6 years) and fifteen healthy elderly donors (age range, 65 to 101 years, mean ± SD, 78 ± 7 years) were selected on the basis of HLA-A*0201 expression and CMV seropositivity. Although elderly donors were not selected according to the SENIEUR criteria, all individuals studied were in good clinical condition. Peripheral blood mononuclear cells (PBMC) were obtained by centrifugation over Histopaque-1077 (Sigma, St Louis, MO, USA). After washing, PBMC were resuspended in phosphate-buffered saline (PBS) and used for flow cytometry analysis.

### Reagents and Monoclonal Abs

For multiparametric flow cytometry the following monoclonal antibodies (mAbs) were used: peridinin chlorophyll protein (PerCP)-conjugated anti-CD8 (SKI); phycoerythrin (PE) conjugated anti-CCR7 (2H4); fluorescein isothiocyanate (FITC) conjugated anti-CD45RA (L48); FITC- and allophycocyanin (APC)-conjugated anti-CD27 (L128); PE- and APC-conjugated anti-CD28 (CD28.2); FITC-conjugated anti-CD244 (C1.7) and FITC-conjugated anti-CD85j (GHI/75). Isotype controls labeled with the different fluorochromes were used in all the experiments. All mAbs were purchased from BD Biosciences (San Jose, CA). The HLA-A*0201/CMV pp65_495–504_(NLVPMVATV) pentamer (A2/CMV-pp65), APC-conjugated, was purchased from Proimmune (Oxford, UK).

### Cell surface staining and flow cytometry

For cell surface staining, 2–2.5 × 10^6 ^PBMC were incubated with A2/CMV-pp65 pentamer, labelled with APC. Staining was performed according to the specifications given by the manufacturer. Cells were then washed and stained with the appropriate combination of mAbs specific for CD8, CCR7, CD45RA, CD27, CD28, CD244 and CD85j for 30 min at 4°C. Subsequently, cells were washed twice with PBS and resuspended in FACS buffer. Flow cytometric analysis was performed on a FACScalibur and FACSCanto cytometers (BD Biosciences). Viable cells were selected using forward and side scatter characteristics. The frequency of A2/CMVpp65 pentamer-positive cells was referred to the CD8^bright ^T cell population. The expression of the different markers was referred to the A2/CMV-pp65 pentamer-positive T cells or to the pentamer-negative CD8^bright ^T cells as indicated. Resulting profiles were analysed using Cell Quest software (BD Biosciences).

### Statistical analysis

SPSS for Windows version 11.5 (SPSS Inc., Chicago) was used for statistical analysis. Comparisons between young, middle aged, and elderly donors were done by analysis of sample normality and variance followed by post hoc multiple comparisons. The tests of Hochberg or Games-Howell were applied for samples with equal or unequal variances respectively. P values of less than 0.05 were considered statistically significant. Data are presented as diagrams of boxes; the lower boundary of the box indicates the 25th percentile and the upper boundary the 75th percentile. Error bars above and below the box indicate the 90th and 10th percentiles. A line within the box marks the median. Outliers are represented as individual points.

## Competing interests

The authors declare that they have no competing interests.

## Authors' contributions

MLP helped design the study, carried out the flow cytometry studies and helped draft the manuscript, in contribution to work included in her PhD thesis. IG performed and analysed some of the flow cytometry. ODR participated in the design of the study and analysis of the flow cytometry data. EMG performed the statistical analysis. CA contributed to the selection of the donors and discussion. RT&JGC participate in the discussion of the results and in the writing of the manuscript. RS conceived the study, participated in its design and coordination, and draft the manuscript. All authors read and approved the final manuscript.
